# Heparanase induced by advanced glycation end products (AGEs) promotes macrophage migration involving RAGE and PI3K/AKT pathway

**DOI:** 10.1186/1475-2840-12-37

**Published:** 2013-02-26

**Authors:** Qiaojing Qin, Jianying Niu, Zhaoxia Wang, Wangjie Xu, Zhongdong Qiao, Yong Gu

**Affiliations:** 1 Department of Nephrology, Shanghai Fifth People’s Hospital, Fudan University, Shanghai 200240, China; 2 School of Life Science and Biotechnology, Shanghai Jiaotong University, Shanghai 200240, China; 3 Department of Nephrology, Huashan Hospital, Fudan University, Shanghai 200240, China

**Keywords:** Advanced glycation end products, Macrophage migration, Diabetes, RAGE, Heparanase, PI3K/AKT

## Abstract

**Background:**

Advanced glycation end products (AGEs), inflammatory-associated macrophage migration and accumulation are crucial for initiation and progression of diabetic vascular complication. Enzymatic activity of heparanase (HPA) is implicated strongly in dissemination of metastatic tumor cells and cells of the immune system. In addition, HPA enhances the phosphorylation of selected signaling molecules including AKT pathway independent of enzymatic activity. However, virtually nothing is presently known the role of HPA during macrophage migration exposed to AGEs involving signal pathway.

**Methods:**

These studies were carried out in Ana-1 macrophages. Macrophage viability was measured by MTT (3-(4,5-dimethylthiazol-2-yl)-2,5-diphenyltetrazolium bromide) assays. HPA and AKT protein expression in macrophages are analysed by Western blotting and HPA mRNA expression by real time quantitative RT-PCR. Release of HPA was determined by ELISA. Macrophage migration was assessed by Transwell assays.

**Results:**

HPA protein and mRNA were found to be increased significantly in AGEs-treated macrophages. Pretreatment with anti-HPA antibody which recognizes the nonenzymatic terminal of HPA prevented AGEs-induced AKT phosphorylation and macrophage migration. LY294002 (PI3k/AKT inhibitor) inhibited AGEs-induced macrophage migration. Furthermore, pretreatment with anti-receptor for advanced glycation end products (RAGE) antibody attenuated AGEs-induced HPA expression, AKT phosphorylation and macrophage migration.

**Conclusions:**

These data indicate that AGEs-induced macrophage migration is dependent on HPA involving RAGE-HPA-PI3K/AKT pathway. The nonenzymatic activity of HPA may play a key role in AGEs-induced macrophage migration associated with inflammation in diabetic vascular complication.

## Introduction

Advanced glycation end products (AGEs), final products of the non-enzymatic reaction between reducing sugars and amino groups in proteins, lipids and nucleic acids, promotes inflammation to accelerate the progression of vascular disease in patients with diabetes as well as other mechanisms [1]. Inflammatory-associated macrophage migration and accumulation in inflamed tissue sites are implicated in the major pathogenic process of vascular complications in diabetes [2-4]. Although the accumulation of advanced glycation end products (AGEs), chronic inflammation-associated macrophage migration and accumulation play critical roles in vascular complication development of diabetes [5-7], knowledge regarding the relationship between AGEs and macrophage migration through extracellular matrix is still unclear.

Heparanase (HPA), an endo-β-glucuronidase, is strongly implicated in cell dissemination associated with tumor metastasis and inflammation. It can cleave heparan sulfate side chains of heparan sulfate proteoglycans to participate in extracellular matrix remodeling and regulate the release of many heparan sulfate-bonded molecules include inflammatory cytokines [8-10]. Moreover, HPA has non-enzymatic activities which play a part in different signaling cascades and selected protein kinase activation associated with cell migration [11,12]. Evidences have shown that over-expressed HPA in most human cancers allow them to penetrate the endothelial cell layer and basement membrane to invade target organs [13,14]. Increased expression of HPA is essential for the development of microvascular complication such as diabetic nephropathy in mice and associated with inflammation in human atherosclerosis [15-17].

Recently, several reports have indicated that AGEs increased HPA expression to facilitate migration of cell associated with inflammation in adult tubular and endothelial cells [18-20]. However, it is unknown whether macrophage migration is induced by AGEs in HPA-dependent manner.

Given the crucial role of AGEs and macrophage migration in the progression of diabetic complications, we thoroughly investigated the effect of AGEs on macrophage migration via HPA independent of enzyme activity. In particular, we analyzed: the effects of AGEs on the mRNA, protein and secretion of HPA; the signaling pathways involved; the effect of an altered HPA expression on macrophage migration and the mechanisms.

## Materials and methods

### Materials

RPMI 1640 and fetal bovine serum (FBS) were from GibcoTM Invitrogen Corporation (Grand Island, NY). Advanced glycation end products (Glycated bovine serum albumin) was from Shanghai Yixin Bio-Technology Co.Ltd (Shanghai, China). RevertAid First Strand cDNA Synthesis Kit was from Fermentas International Inc (Graiciuno, Vilnius, Lithuania). Real time PCR Master Mix was from Delaware Biotechnology Institute (Newark,DE). SuperECL Plus and LY 294002 were from Beytime Institute of Biotechnology (Haimeng, China). ELISA kit for mouse HPA was from Glory Science Co,Ltd (Hangzhou, China). Rabbit anti-mouse HPA, RAGE, AKT antibody and peroxidase-labeled goat anti-rabbit second antibody were from Wuhan Boster Bio-engineering Limited Company (Wuhan, China). Rabbit anti-mouse GAPDH antibody was from Santa Cruz Biotechnology Inc (Santa Cruz, CA); Rabbit anti-mouse phospho-AKT antibody was from Cell Signaling Technology (Boston, MA). MTT was from Sigma-Aldrich (Shanghai, China). Falcon™ cell culture insert system was from Becton Dickinson and Company (Franklin Lakes, NJ).

### Cell culture

Ana-1 mouse macrophage cell line was obtained from the cell bank of Shanghai Institutes for Biological Sciences, Chinese Academy of Sciences (Shanghai, China). Cells were maintained in RPMI 1640 medium supplemented with 10% fetal bovine serum and 100 units/ml penicillin and 100 μg/ml streptomycin and were incubated at 37°C in 5% CO_2_ humidified air. Spent medium was replaced every 2–3 days. Cells were grown to 80% confluence and then serum-starved for 16 hours before use.

### MTT assay

In order to determine the effects and mechanism of AGEs on HPA in macrophages, we performed assays to determine the concentrate of AGEs, LY294002 (PI3k/Akt inhibitor), anti-HPA and RAGE antibody which didn’t change the viability of macrophages significantly. 100 μl macrophages were seeded at a density of 5 × 10^4^ cells/ml and incubated with AGEs, LY294002, anti-HPA and RAGE antibody at the indicated concentration in 96-well plates. After 24 h incubation, 3-(4,5-dimethyl-thiazol-2-yl)-2,5-diphenyltetrazolium bromide (MTT) solution was added to each well for 4 hours. Finally, the blue salt in each well was dissolved and the plates were read by using a microplate reader with RPMI 1640 as blank and cell culture medium as control. The results of cell viability determined 100 mg/L AGEs, 15 μM LY294002, 10 μg/ml anti-RAGE and 10 μg/ml anti-HPA antibody in the following experiments (The choice of dosage is showed in results and discussion).

### Treatments with cells

First, we evaluate the role of RAGE, HPA, AKT in AGEs-induced macrophage migration. Cells were treated with AGEs (100 mg/L), and LY294002 (pre-treated with 15 μM LY294002), and anti-RAGE or HPA antibody (pre-treated with 10 μg/ml anti-RAGE or HPA antibody) for 24 h. Subsequently, cells were treated with AGEs (100 mg/L), and anti-RAGE antibody (pre-treated with 10 μg/ml anti-RAGE antibody) for 24 h and analyzed by RT-PCR, Western blot and ELISA assay to examine effects of AGEs on HPA expression and the role of receptor for advanced glycaiton end products (RAGE) in AGEs-induced HPA expression. Finally, we treated cells with AGEs (100 mg/L), and anti-RAGE or HPA antibody (pre-treated with 10 μg/ml anti-RAGE or HPA antibody) for 24 h, and harvested for Western blot analysis to assess of the role of HPA and RAGE in AGEs-induced activation of AKT.

### Migration assay

Macrophages used for seeding transwell inserts were harvested from the plate by incubating as above and plated into transwell inserts with 8 μm pores, which were coated with a thin layer of Matrigel and placed in a 6-well plate. 2% FBS were added to the lower chamber. Plates were incubated for 6 h at 37°C. Cells remaining on the upper membrane surface were removed with a cotton swab. Upper wells were placed into 4% paraformaldehyde for 15 min to fix cells adherent to the underside of the membrane. Migrated cells were stained with hematoxylin and counted (6 random fields per slide) in ten 40× fields.

### ELISA for HPA

The Ana-1 macrophages were plated at 5 × 10^5^ cells/well in 24 well plates overnight. Macrophages were cultured separately with 100 mg/L AGEs, and anti-RAGE antibody (pre-treated with 10 μg/ml anti-RAGE antibody) for 24 h. The levels of HPA in culture supernatants were determined using commercially available enzyme linked immunosorbent assay (ELISA) kits according to the manufacturer’s instructions. Briefly, 10 μl supernatant and 40 μl sample diluent was added to sample well, followed by the addition of 100 μl HRP-conjugate reagent to each well and incubated for 60 minutes at 37°C. The reaction was visualized by the addition of 50 μl chromogen solution A and 50 μl chromogen solution B for 15 min at 37°C. The reaction was stopped with 50 μl stop solution and absorbance at 450 nm was measured using ELISA plate reader within 15 min. The level of HPA was quantified by a standard curve established by a serial dilution of standard concentration.

### Detection of mRNAs by real time quantitative RT-PCR

RNA from Ana-1 macrophages was isolated using TRIZOL reagent following the manufacturer’s instruc-tions. Isolated RNA was reversed transcribed into complementary DNA using RevertAid First Strand cDNA Synthesis Kit according to the protocol by the manufacturer. To determine the fold-changes in the expression of HPA gene, real-time PCR was performed using the first strand cDNA, the forward and reverse primers (forward: GAACCTCCATAATGTCACCAAGC, resverse: GTCTGCTCATCCACCATCTTCAG), and Bestar™ Taqman Real time PCR master mix. Thermocycling conditions for SYBR Green consisted of a denaturation step for 2 min at 95°C followed by 35 cycles of 95°C for 15 s, 59°C for 30 s and 72°C 15 s. Data analysis was performed using standard curve method and ΔΔCt method. The mount of HPA was determined and normalized by the amount of GAPDH cDNA.

### Western blot analysis

The cells were lysed with dissociation solution containing phosphatase, protease inhibitors and PMSF. Protein extracted from macrophages was electrophoresed on SDS-polyacrylamide gels (10%) and Western blotted. Proteins were transblotted onto nitrocellulose membrane. Membranes with the transferred proteins were blocked for 1 hour with 5% (w/v) skim milk powder diluted in PBS and then incubated with anti-HPA, AKT or phosphorylated (p)-AKT antibodies (1:1000 dilution) respectively at 4°C overnight. The membranes were incubated with a peroxidase-conjugated secondary antibody respectively and visualized by a supper enhanced chemiluminesence detection system. Densitometric analysis was performed by normalizing band density to that for GAPDH.

### Statistical analysis

The data were expressed as mean ± SEM. Statistical analysis was performed by SPSS software using one-way analysis of variance (ANOVA). A value of P < 0.05 was considered statistically significant.

## Results

### Cell viability

**Figure 1:**
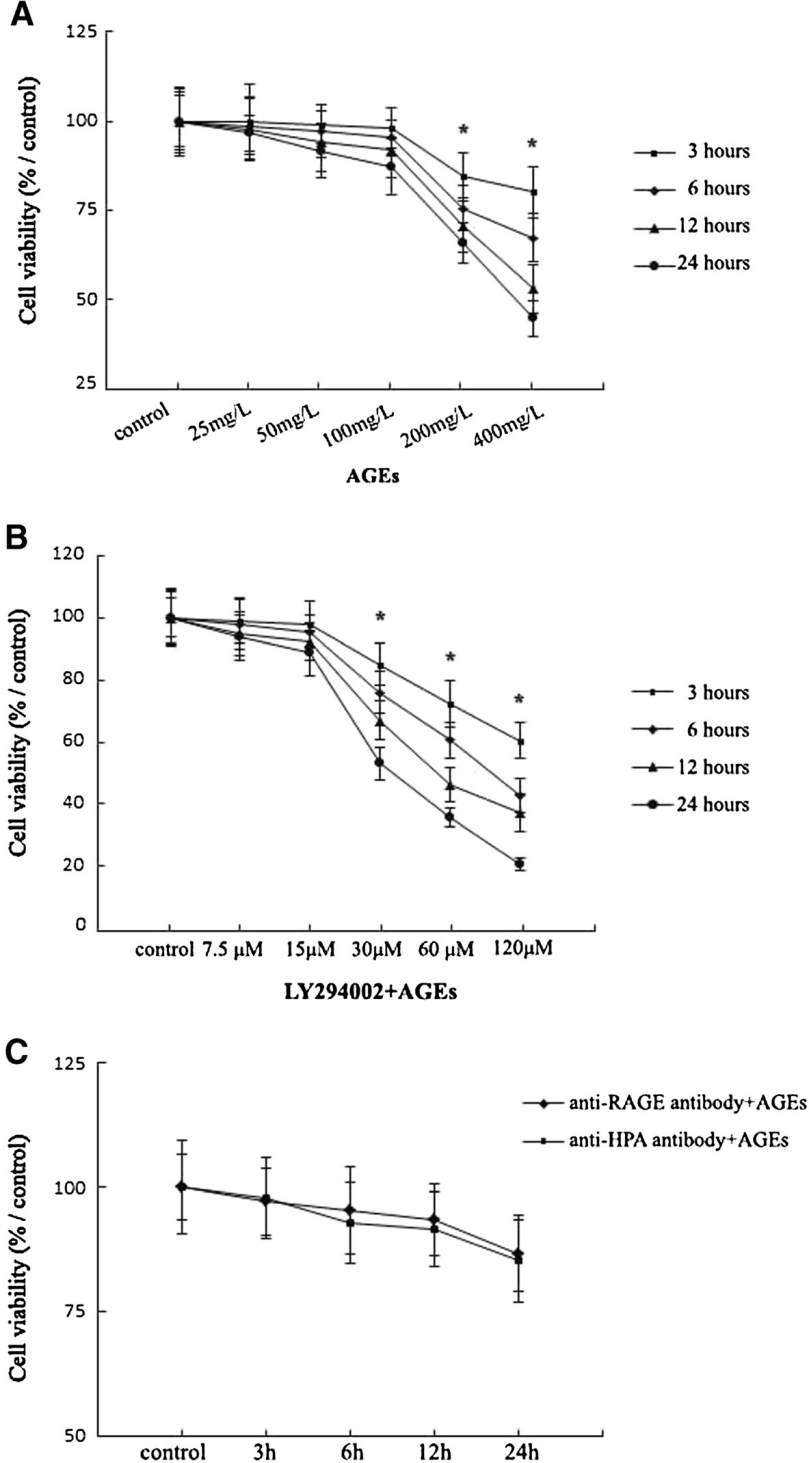
**Viability analysis of Ana-1 macrophages after treatment with AGEs, LY294002, anti-RAGE or HPA antibody.** Cell viability assay is performed using MTT assay. **A**, Cells (5 × 10^4^) were treated with AGEs (0, 25, 50, 100, 200 and 400 mg/L) for 3, 6, 12, 24 h. **B**, Cells (5 × 10^4^) were pretreated with LY294002 (7.5-120 μM) for 1 h before culture with 100 mg/L AGEs for 3, 6, 12, 24 h. **C**, Cells (5 × 10^4^) were pretreated with anti-RAGE or HPA antibody for 1 h before culture with 100 mg/L AGEs for 3, 6, 12, 24 h. The results represent the mean of six culture wells (mean ± SEM). *p < 0.05, as compared to the control group. All of the experiments were performed independently in triplicate.

Incubation of Ana-1 macrophages with AGEs (25–400 mg/L) for 3,6,12 and 24 h led to the significant dose-dependent reduction in the viability of cells compared with controls. AGEs didn’t change significantly the viability of Ana-1 macrophages within 25-100 mg/L AGEs concentration range for 3,6,12 and 24 h incubation (Figure 1A). Therefore, we chose the max dosage of AGEs at 100 mg/L, which didn’t change the viability significantly in Ana-1 macrophages within 24 h incubation, to finish the following experiments involving the mechanism.

Subsequently, macrophages were pre-treated with LY294002 (7.5-120 μM) for 1 h before culture with 100 mg/L AGEs except control group. The treatment of macrophages with LY294002 (7.5-15 μM) and 100 mg/L AGEs within 24 h culture didn’t affect cell viability significantly. Exposure to LY294002 (30–120 μM) and 100 mg/L AGEs for 3, 6, 12, 24 h decreased macrophage viability significantly (Figure 1B). We chose the max dosage of LY294002 at15 μM and 100 mg/L AGEs for 24 h incubation, which didn’t change the viability significantly in Ana-1 macrophages, to finish the following experiments.

In addition, the pre-treatment with 10 μg/ml anti-RAGE or 10 μg/ml anti-HPA antibody in 100 mg/L AGEs-induced macrophages for 24 h culture didn’t change the cell viability significantly (Figure 1C).

### HPA play a key role in the AGEs-induced macrophage migration involving RAGE and PI3K/AKT pathway

**Figure 2:**
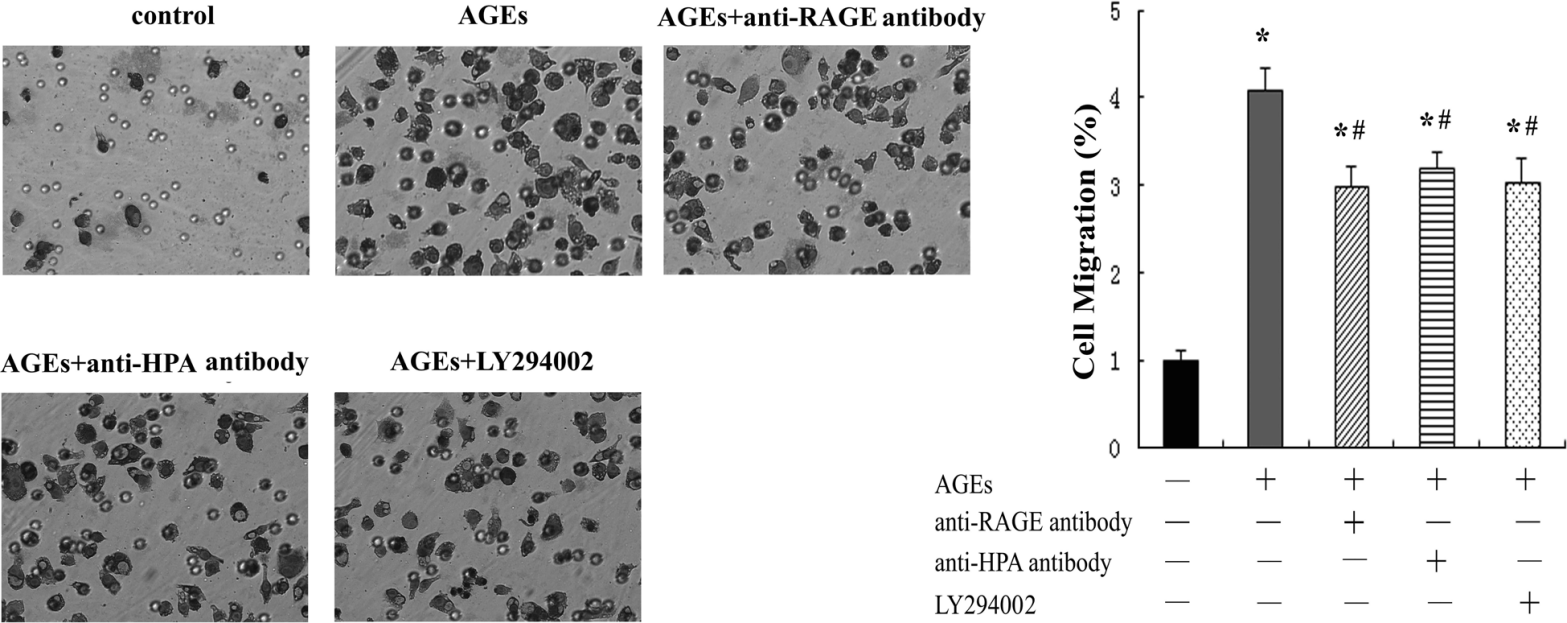
**HPA, RAGE and PI3K/AKT pathway correlate with AGEs-induced macrophage migration.** Cells were cultured with AGEs for 24 h with or without pre-treatment with LY294002, anti-HPA or RAGE antibody for 1 h. The migration was measured by transwell assays. Results were normalized to the number of macrophages that migrated in control group. The results represent the mean of six culture wells (mean ± SEM). *p < 0.05 compared to control and #p <0.05 compared to AGEs. All of the experiments were performed independently in triplicate.

To investigate the effect of AGEs on macrophage migration, we used the cell culture insert system and found more macrophage migration after co-culture of AGEs with macrophages for 24 h. Meanwhile, we performed the changes of the AGEs-induced macrophage migration using anti-HPA antibody which recognizes the COOH-terminal domain, nonenzymatic terminal, to determine the signaling role of HPA in AGEs-induced macrophages infiltration. Our data showed that AGEs-induced macrophage migration was reduced after pretreatment with anti-HPA antibody for 1 h. In addition, pretreatment with anti-RAGE antibody or LY294002 for 1 h decreased the AGEs-induced macrophages migration significantly (Figure 2). The results explore that HPA mediates AGEs-induced macrophage migration and may involve RAGE and AKT pathway.

### AGEs changes HPA mRNA, protein expression and secretion via RAGE in macrophages

**Figure 3:**
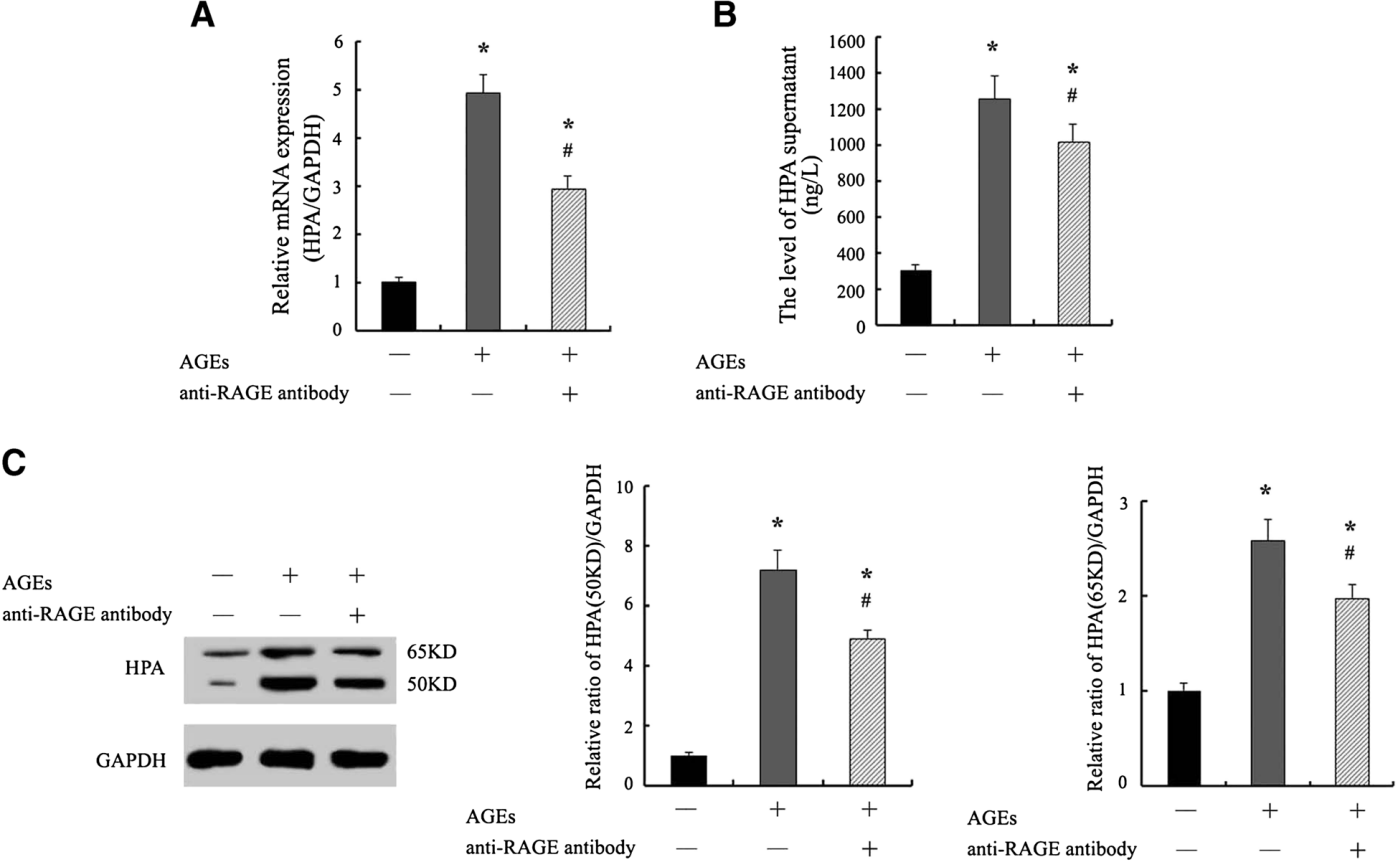
**AGEs up-regulates HPA mRNA, protein expression and secretion in macrophages via RAGE.** Cells were cultured with AGEs for 24 h with or without pre-treatment with antibody against RAGE for 1 h. **A**, The levels of HPA mRNA were assessed with real time quantitative RT-PCR. **B**, The secretion of HPA in supernatant was measured by enzyme-linked immunosorbent assay (ELISA). **C**, The expression of HPA protein in macrophages was determined by Western blotting. The results represent the mean of six culture wells (mean ± SEM). *p < 0.05 compared to control and #p <0.05 compared to AGEs. All of the experiments were performed independently in triplicate.

To test the possible effect and mechanism of AGEs treatment on HPA expression, Ana-1 macrophages were incubated for 24 h in the presence of 100 mg/L AGEs with or without the pretreatment of 10 μg/ml anti-RAGE antibody for 1 h. Using RT-PCR and ELISA assays, we show that AGEs promoted the levels of mRNA and secretion significantly in macrophages. Pretreatment with anti-RAGE antibody significantly inhibited HPA secretion and mRNA production in AGEs-induced macrophages (Figure 3A and 3B).

Furthermore, the HPA latent form (65 kDa) and active form (50 kDa) were identified using an antibody against the C-terminus domain of HPA protein. We show that untreated cells primarily expressed the 65 kDa latent form. Both of 65 kDa and 50 kDa forms were increased significantly and the 50 kDa actived form was more strongly induced as compared with the 65 kDa latent form in AGEs-induced macrophages. Pretreatment with anti-RAGE antibody attenuated AGEs-induced 50 kDa actived and 65 kDa latent forms in macrophages (Figure 3C).

### Phosphorylation of AKT are increased in AGEs-stimulated macrophages via HPA involving RAGE

**Figure 4:**
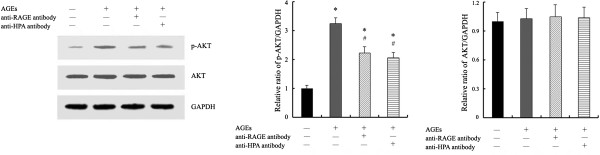
**The expression of AKT protein in AGEs-induced macrophages.** Cells were cultured with AGEs or pretreated with antibody against RAGE or HPA for 1 h before exposed to AGEs for 24 h. AKT and p-AKT protein expression is determined by Western blot analysis using anti-AKT and p-AKT antibody. The results represent the mean of six culture wells (mean ± SEM). ^*^P < 0.05 compared to control and ^#^P <0.05 compared to AGEs. All of the experiments were performed independently in triplicate.

We examine the expression of AKT protein in macrophages to explain the role of PI3K/AKT signaling pathway in AGEs-induced macrophage migration via HPA. We found that the phosphorylation of AKT was increased significantly at 24 h of stimulation with AGEs in macrophages. The expression of AKT phosphorylation could be inhibited by the pretreatment of an antibody recognizing the COOH-terminal domain of HPA protein or anti-RAGE antibody in AGEs-induced macrophages. Furthermore, we didn’t find the changes of total AKT protein in macrophages with different treatment (Figure 4).

## Discussion

Cardiovascular complications are the leading cause of death in patients in diabetic patients [21,22]. The current study provides evidence to support that excess accumulation of advanced glycation end products (AGEs) and inflammation is emerging as important mechanism for micro- and macrovascular complication of diabetes [23,24]. Monocytes transmigrate into the subendothelial space and differentiate into macrophages, which migrate, infiltrate and accumulate in the vascular tissues, involving in diabetic vascular complications. Clinical study has also observed significant increase of AGEs accumulation in diabetic vascular tissues [25], which may induce macrophage migration across an endothelial cell monolayer [26].

Heparanase (HPA), a mammalian endo-beta-D-glu-curoxnidase, has previously been shown to be a key enzyme in the metastatic potential of tumor-derived cells and cells of the immune system [27-29]. Recently, evidences show that HPA enhances the phosphorylation of selected signaling molecules, in a manner that is mediated by its C-terminal domain but not enzymatic activity [12,30]. In the study, we have characterized the role and mechanism of HPA in AGEs-induced macrophage migration independent of enzymatic activity.

With the MTT assay we observed the dose-dependent reduction of viability in macrophages treated by AGEs. The viability revealed a nonlinear dose response to AGEs in macrophages. AGEs treatment at 25, 50 and 100 mg/L for 3,6,12 and 24 h incubation didn’t result in a significant loss of viability in macrophages compared with controls (0 mg/L) (Figure 1A). We then used 100 mg/L AGEs, a max dosage which didn’t changed the viability significantly within studied AGEs concentration range at 24 h culture, for further studies on the role of HPA in macrophages.

Moreover, pretreatment with LY294002 (7.5-15 μM), anti-RAGE or HPA antibody (10 μg/ml) for 1 h before culture with 100 mg/L AGEs for 3,6,12 and 24 h incubation didn’t result in a significant loss of viability in macrophages compared with controls (Figure 1B). We then used preincubation of 15 μM LY294002, 10 μg/ml anti-RAGE or HPA antibody, the dosage which didn’t changed the viability significantly within the studied concentration range at 24 h culture, for further studies in 100 mg/L AGEs-induced macrophages.

Evidences have shown that HPA play a role in signaling pathway [31]. C-terminus domain mediates nonenzymatic functions of HPA, facilitating the phosphorylation of phosphatidylinositol 3-kinase/protein kinase B (PI3K/AKT), in an enzymatic activity-independent manner in pituitary tumor and proximal tubular [32,33]. Recent studies show that AKT (protein kinase B), a serine/threonine protein kinase, regulates monocyte/macrophage migration. Inhibition of AKT pathways decreased macrophage migration while mammalian cell migration can be promoted by enhancing AKT signaling [34-36].

In agreement with previous study [26,37], we have shown that AGEs induced macrophage migration significantly. However, more importantly, we have shown that pretreatment of anti-HPA antibody, which bind to the C-terminus domain of HPA specially, inhibited the macrophage migration significantly (Figure 2). This demonstrates that C-terminus domain of HPA mediates AGEs-induced macrophage migration. We speculate that the C-terminus domain of HPA may mediate AGEs-induced macrophage migration via AKT signaling pathway.

Subsequently, we detected the role of PI3K/AKT signaling pathway in AGEs-induced macrophage migration via HPA. AKT is a target of PI3K activation and its phosphorylation is prevented by PI3K/AKT inhibitors. Although AKT phosphorylation is increased by external HPA in a short time, the AKT phosphorylation induced by changes of HPA protein after AGEs treatment may need longer time. So we chose to determine the levels of AKT at 24 h culture. The results show that the levels of AKT phosphorylation was increased at 24 h in macrophage cultured with AGEs. Pretreatment with an antibody recognizing the C-terminus domain of HPA protein inhibited AKT phosphorylation significantly (Figure 4). The results suggest that AGEs could activate PI3K/AKT signaling pathway via C-terminus domain of HPA protein. Furthermore, we observed that AGEs-induced cell migration was attenuated by using LY294002, a PI3K/AKT inhibitor, in macrophages (Figure 2). These data indicate that the AGEs-induced macrophage migration is partially mediated by the activation of PI3K/AKT signaling pathway in HPA-dependent manner. HPA-PI3K/AKT signal pathway may be important in macrophage migration induced by AGEs.

Recently, it was reported that AGEs could induce HPA expression through receptor for advanced glycation end products (RAGE) in human microvascular endothelial cells [19]. Substantial evidence has demonstrated that RAGE plays a central role in the etiology of diabetes complications and inflammation [38-40]. RAGE ligand, such as HMGB-1, could stimulate phosphorylation of AKT and cell proliferation/migration through RAGE/PI3K/AKT signal transduction pathway [41,42]. Here we assess the role of RAGE on HPA expression and AKT pathway which associated with macrophage migration.

We observed that treatment with AGEs for 24 h culture resulted into significant increase of HPA mRNA, HPA latent form (65 kDa) and enzyme form (50 kDa) compared with untreated cells. 50 kDa enzyme form is more strongly induced than 65 kDa latent form. All the changes could be attenuated by pretreatment with anti-RAGE antibody (Figure 3A and 3C). These results show that increased expression of HPA mRNA and protein are associated with AGEs stimulation via RAGE and HPA protein expression may partly dependent of post-transcriptional regulation in macrophages.

Furthermore, ELISA analysis was employed to determine the levels of HPA in supernatant. We discovered that pretreatment with anti-RAGE antibody inhibited the increased secretion of HPA in AGEs-stimulated macrophages significantly (Figure 3B). The result from ELISA suggests that AGEs could induce HPA protein secretion via RAGE as well as the results from Western blot. These data provide the first evidence for AGEs-induced macrophage HPA mRNA, protein expression and secretion in a RAGE-dependent manner. RAGE-HPA pathway may play a key role in AGEs-induce macrophage migration.

To analyze the relation between RAGE, AKT phosphorylation and migration to further demonstrate RAGE-HPA-PI3K/AKT pathway, an antibody against RAGE is used to block the effect of RAGE in AGEs-induced macrophages. The results show that pretreatment of the cells with the blocking antibody to RAGE suppressed AGEs-induced AKT phosphorylation (Figure 4) and cell migration significantly in macrophages (Figure 2). The result suggests that RAGE could mediate AGEs-induced macrophage migration via AKT pathway and agrees to RAGE-HPA-PI3K/AKT pathway associated with AGEs-stimulated macrophage migration.

## Conclusions

Our results show for the first time that AGEs-induced macrophage migration may be mediated by RAGE-HPA-PI3K/AKT signal pathway. It supports the notion that HPA could mediate macrophage migration involving in RAGE-HPA-PI3K/AKT pathway independent of enzymatic activity, providing new insights into the role of HPA in AGEs-induced macrophage migration associated with vascular complication of diabetes. The mechanism such as regulation of HPA expression and AKT phosphorylation mediated by HPA might be required for further studies.

## Abbreviations

AGEs: Advanced glycation end products; RAGE: Receptor for advanced glycation end products; HPA: Heparanase; PI3K/AKT: Phosphatidylinositol 3-kinase/protein kinase B.

## Competing interests

The authors declare that they have no competing interests.

## Authors’ contributions

QQ carried out the design, analysis and writing of the manuscript. YG participated in its design and coordination and helped to draft the manuscript. ZW, JN, WX and ZQ participated in conduct and analysis. All authors read and approved the final manuscript.
